# Association of extracellular vesicle inflammatory proteins and mortality

**DOI:** 10.1038/s41598-022-17944-z

**Published:** 2022-08-18

**Authors:** Nicole Noren Hooten, Stephanie Torres, Nicolle A. Mode, Alan B. Zonderman, Paritosh Ghosh, Ngozi Ezike, Michele K. Evans

**Affiliations:** 1 Laboratory of Epidemiology and Population Science, National Institute on Aging, National Institutes of Health, 251 Bayview Boulevard, Baltimore, MD 21224 USA; 2 Present Address: Edward Via College of Osteopathic Medicine at University of Louisiana Monroe, Monroe, LA USA; 3 Laboratory of Clinical Investigation, National Institute on Aging, National Institutes of Health, 251 Bayview Boulevard, Baltimore, MD 21224 USA

**Keywords:** Diagnostic markers, Mechanisms of disease

## Abstract

Even before the COVID-19 pandemic declines in life expectancy in the United States were attributed to increased mortality rates in midlife adults across racial and ethnic groups, indicating a need for markers to identify individuals at risk for early mortality. Extracellular vesicles (EVs) are small, lipid-bound vesicles capable of shuttling functional proteins, nucleic acids, and lipids. Given their role as intercellular communicators and potential biomarkers of disease, we explored whether circulating EVs may be markers of mortality in a prospective, racially, and socioeconomically diverse middle-aged cohort. We isolated plasma EVs from 76 individuals (mean age = 59.6 years) who died within a 5 year period and 76 surviving individuals matched by age, race, and poverty status. There were no significant differences in EV concentration, size, or EV-associated mitochondrial DNA levels associated with mortality. We found that several EV-associated inflammatory proteins including CCL23, CSF-1, CXCL9, GDNF, MCP-1, STAMBP, and 4E-BP1 were significantly associated with mortality. IL-10RB and CDCP1 were more likely to be present in plasma EVs from deceased individuals than in their alive counterparts. We also report differences in EV-associated inflammatory proteins with poverty status, race, and sex. Our results suggest that plasma EV-associated inflammatory proteins are promising potential clinical biomarkers of mortality.

## Introduction

There are significant long standing health disparities in the United States (US). One of the most glaring, and disturbing gaps lie in the life expectancy of different racial and ethnic groups. For example, African American individuals living below poverty are particularly vulnerable to early death compared to their White counterparts even with adjustments for various lifestyle factors^1,2^. Low socioeconomic status remains a significant risk factor for morbidity and premature mortality^3^. The COVID-19 pandemic has highlighted alarming health disparities in mortality among minority populations^4,5^ . Recent data in the US also show a disturbing increase in midlife mortality rates across racial-ethnic populations^6^. These reported changes in mortality in adults aged 25–64 years contribute to an overall decline in life expectancy in the US noted in the pre-COVID-19 period, which has led to the US ranking the lowest in life expectancy among the high income developed countries^7^.

Identifying individuals at risk for early mortality would aid interventions and treatment for these populations^8^. Recent advances in the characterization of extracellular vesicles (EVs) suggest that these biological structures may be indicators of health and disease^9,10^. EVs are lipid-encapsulated nano-sized membranous particles that are secreted from cells into the extracellular space including biofluids. Circulating EVs can be reliably isolated from plasma and serum making them ideal candidates for biomarkers^11,12^. However, we are only beginning to understand and characterize EVs in human populations.

The general term EVs comprises vesicles from three main categories including exosomes, microvesicles and apoptotic bodies^10,13^. However, the overlap in physical properties and also the difficulty in determining a particular biogenesis pathway has led to collectively referring to these particles as EVs^14^. EVs carry bioactive cargo including nucleic acids (microRNAs, mRNAs, DNAs), lipids and proteins^15–18^. This cargo can be delivered to recipient cells to elicit functional consequences^19,20^. In this regard, EVs can regulate a myriad of biological processes depending on the cellular context and stimuli^10^. Emerging evidence suggests that the molecular information contained in EVs can be harnessed for diagnostic and therapeutic purposes^9,21^. Therefore, EVs have been proposed as biomarkers of various age-related diseases including cancer^9^, neurodegenerative diseases^22^, cardiovascular disease^23^ and diabetes mellitus^24^.

Previously, we examined EV characteristics and protein cargo in a racially diverse population of middle-age adults^25^. In this cohort, EV concentration was associated with several well-established clinical markers of mortality. These markers included high-sensitivity C-reactive protein, homeostatic model assessment of insulin resistance (HOMA-IR), alkaline phosphatase, body mass index, waist circumference and pulse pressure^25^. EV protein cargo was also associated with mortality markers and this association was dependent on race. These data led us to further explore whether EVs and their associated cargo can be utilized as novel clinical markers of mortality.

## Results

### Plasma EV characteristics of mortality cohort

In order to examine whether EVs may be markers of mortality, we identified deceased individuals within the HANDLS cohort whose cause of death was due to either cardiovascular disease or cancer, which are the most common causes of deaths for HANDLS participants^26^ and the US nationally^27^. African American and White participants aged 45–74 were included in the study who had donated a sample within five years of their death (n = 76) and were matched to participants who remained alive through 2017 (n = 76; Table 1). Details on matching criteria are found in Materials and Methods and demographic information is included in Table 1.

**Table 1:** Demographics for EV and mortality sub-cohort of the HANDLS study.

Characteristics	Alive	Died	*P*-Value
N	76	76	
Age (mean (SD))	59.62 (6.16)	59.46 (6.17)	0.872
Male (%)	39 (51.3)	39 (51.3)	1
African American (%)	40 (52.6)	40 (52.6)	1
Below poverty (%)	36 (47.4)	36 (47.4)	1

Pearson’s chi-squared tests were used to analyze differences for sex, race and poverty status. Student’s t-test was used to analyze differences among the groups for age. SD = standard deviation.

**Figure 1:**
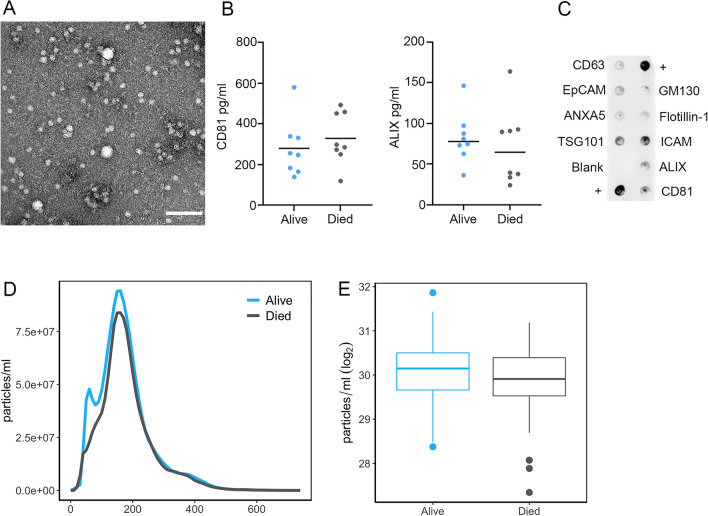
EV characteristics of mortality cohort. (**A**) EV morphology and size were visualized using electron microscopy (scale bar = 200 nm). (**B**) Plasma EVs from alive (n = 8) and individuals who had died (n = 8) within 5 years of sample collection were lysed and the EV markers CD81 and ALIX using quantified using ELISA assays. Individual data points are shown, and the bar represents the mean. There were no significant differences in CD81 and ALIX EV levels between alive and deceased individuals using Student’s t-test. (**C**) EV markers in plasma EVs were assessed using an Exo-Check™ Exosome Antibody Array. (**D,E**) Plasma EVs were isolated from participants who were alive or died within five years of sample donation (Table 1), and EV size distribution and concentration were analyzed by Nanoparticle Tracking Analysis. (**D**) Size distribution was averaged for each group (n = 75 for alive and n = 74 for died groups). EV concentration is shown in the plots in (**E**).

We isolated EVs from plasma samples of the 76 alive and 76 deceased individuals. We chose to separate our EVs using a precipitation approach as we have previously reported that this technique is reproducible and suitable for isolation from a large number of clinical samples^28^. Isolated plasma EVs were validated according to Minimal Information for Studies of Extracellular Vesicles (MISEV) guidelines from the International Society of Extracellular Vesicles^29^. Electron microscopy was used to visualize EVs and showed images of round, intact vesicles in the expected morphology of EVs (Fig. 1A). EVs were lysed and the presence of the known EV markers, CD81 and ALIX, were verified using an ELISA assay (Fig. 1B). EV markers were also assessed using an Exo-Check™ Exosome Antibody Array. The presence of ALIX and CD81 in our EV preparations were further validated using the array along with other EV markers including TSG101 and ICAM, and at a lower level CD63, EpCAM, ANXA5, and Flotillin-1 (Fig. 1C). GM130 was included to assess the purity of the isolated EVs. Nanoparticle tracking analysis (NTA) confirmed a size distribution of vesicles between 50 and 350 nm, which is typical of EVs isolated from plasma (Fig. 1D). Combined these data validate that our plasma EVs have a typical morphology, size and protein markers that are characteristic of EVs.

We examined whether there were changes in plasma EV concentration in participants who had died within 5 years compared to those who were alive. NTA was used to calculate plasma EV concentration. Although mean EV concentration was lower in individuals who died within five years, this difference was not significant (*P* = 0.09; Fig. 1E). In this cohort, there were also no significant differences in EV concentration with race, sex, or poverty status.

### EV-associated mtDNA levels and mortality

Previously, we reported that circulating cell-free mitochondrial DNA (ccf-mtDNA) is present in EVs and that EV-plasma levels decline with advancing age^28,30^. As ccf-mtDNA can act as a damage-associated molecular pattern (DAMP) eliciting immune and inflammatory responses, we posited that there may be differences in mtDNA cargo in EVs from the alive cohort compared to those in the mortality cohort. DNA was purified from plasma EVs and mtDNA was quantified using primers that were designed against four different regions of the mitochondrial genome (Supplementary Table 1). The primers were designed against areas crossing the 16S rRNA and tRNA-Ile1 regions (Mito_3164), the NADH dehydrogenase 2 (ND2) region (Mito_4625), the Cytochrome c oxidase subunit 2 (*COX2*) region (Mito_7878) and the ATP8 gene region (Mito_8446)^30^. These four primer sets were used to amplify these different mitochondrial regions using quantitative real-time PCR (qPCR).

**Figure 2:**
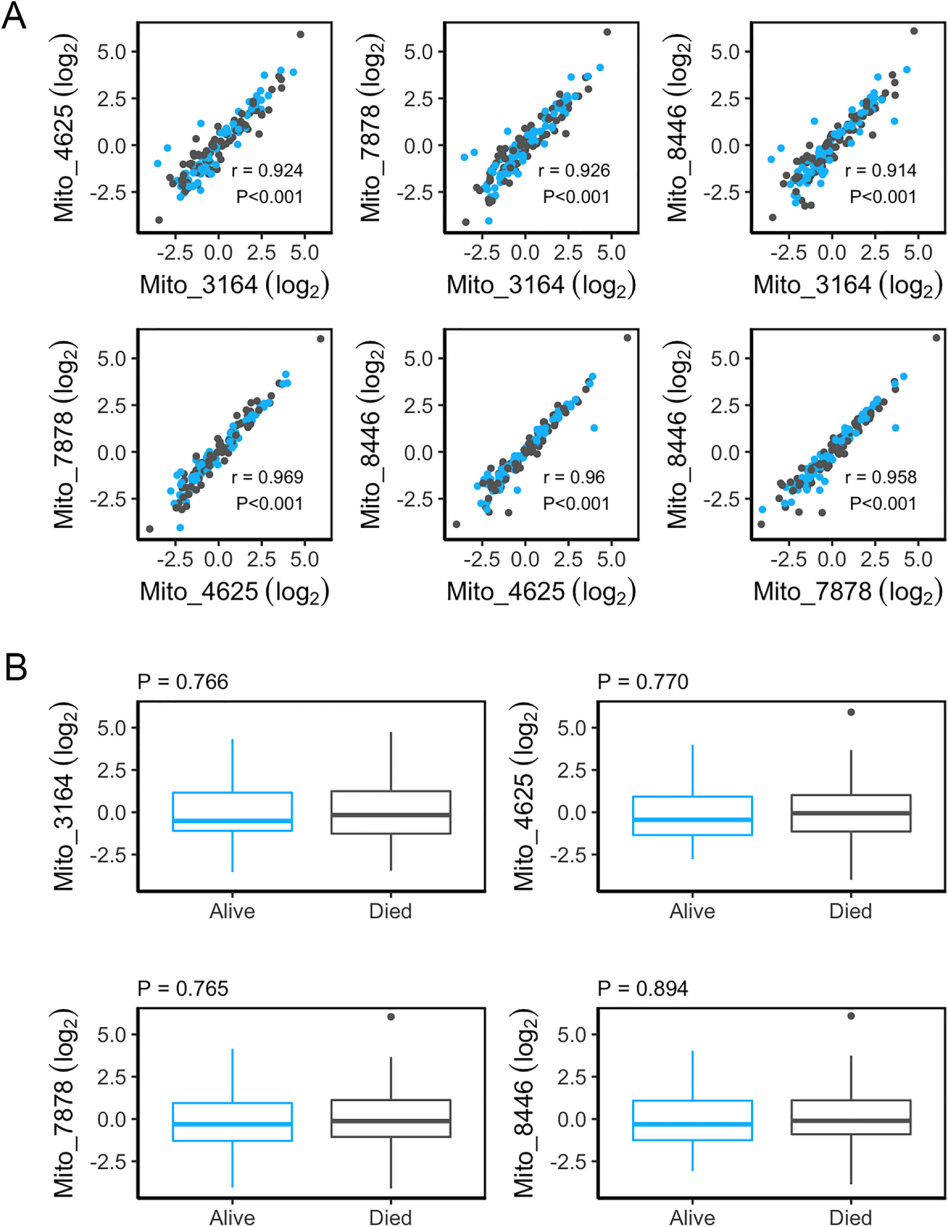
Association of mtDNA levels and mortality. (**A**) Plasma EVs were isolated from 76 individuals deceased within 5 years of sample donation along with 76 surviving individuals. DNA was isolated and EV mtDNA levels (log_2_ transformed) were measured using mtDNA specific primers from four different regions of the mitochondrial genome using qPCR. Pearson correlation r and *P* values are indicated for each primer pair. (**B**) Linear regression was used to determine the relationships between mtDNA levels (log_2_ transformed) and mortality status accounting for sex, race, and poverty status. There were no significant differences.

We analyzed the DNA isolated from plasma EVs from our mortality cohort. Initially, we examined the relationship between the mtDNA amplicons from each of the four primer sets. Pearson correlation showed a significant positive relationship in the mtDNA levels amplified by each primer set (Fig. 2A). Next, we analyzed the relationship between the EV mtDNA levels amplified from each of the different primer sets in this cohort and mortality status. We found that there was no significant association between EV mtDNA levels and mortality status (Fig. 2B).

### Plasma EV mtDNA levels are not associated with EV concentration

To determine whether differences in EV mtDNA levels may be due to changes in EV concentration, we examined this relationship. There was no significant association between EV concentration and mtDNA levels for each of the four primer sets (Supplementary Fig. 1). These data are consistent with our previous results showing a lack of a relationship between these variables in an aging cohort^30^.

### Plasma EV inflammatory proteins are associated with mortality

**Figure 3:**
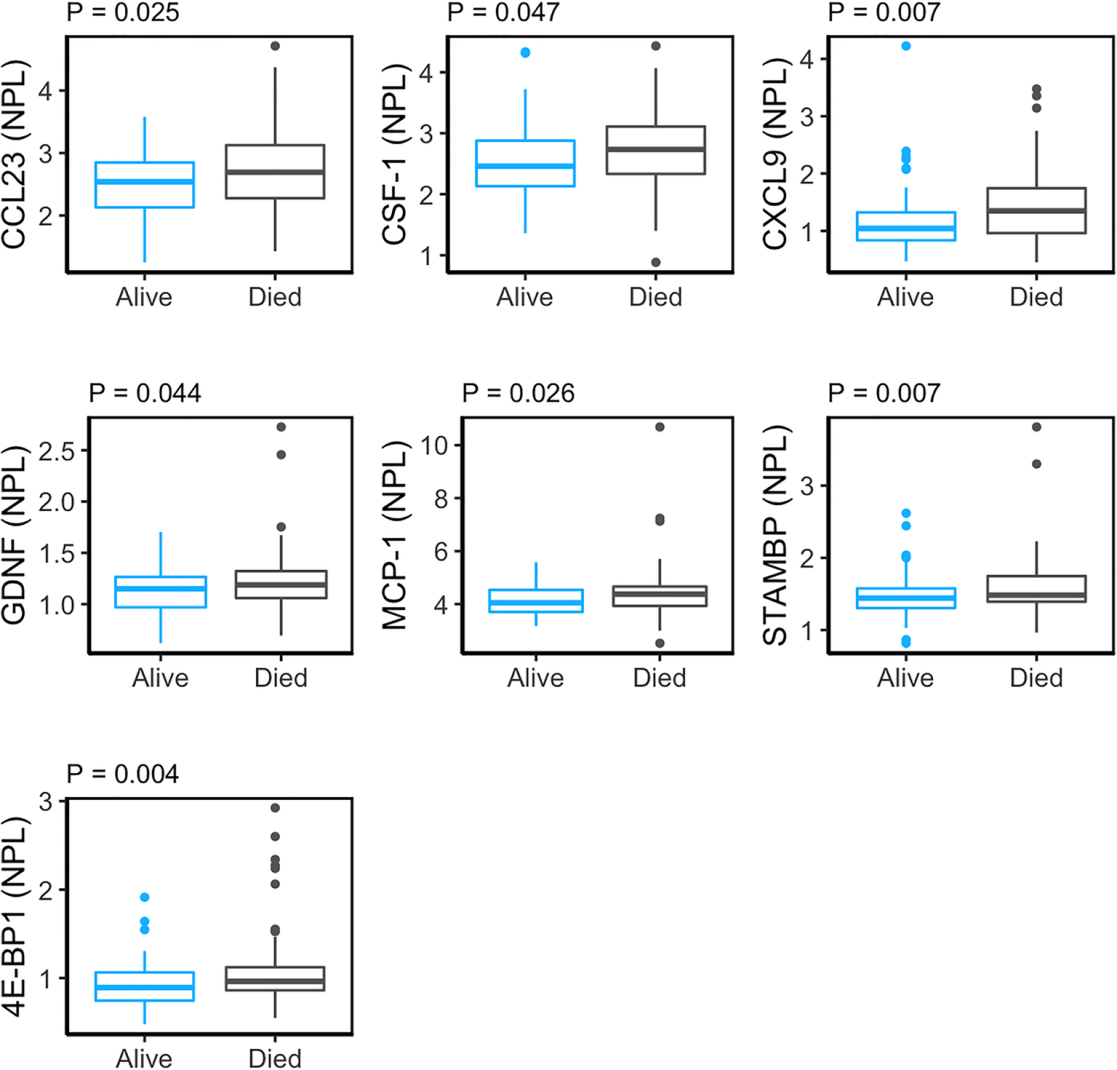
Significant association of EV inflammatory protein levels with mortality. Plasma EVs were isolated from 76 individuals deceased within 5 years along with 76 surviving individuals (Table 1). EVs were lysed and analyzed using a proximity extension assay. Normalized protein levels (NPL) are shown. Linear regression was used to determine the relationships between protein levels and later mortality status accounting for sex, race, and poverty status.

We further tested whether other EV-associated cargo may be associated with mortality status. We focused on inflammatory proteins in EVs as we have previously shown that EV inflammatory proteins are altered with diabetes mellitus status and disease severity^31^. In order to test this, we lysed our plasma EVs and quantified EV-associated inflammatory proteins using Multiplex Proximity Extension Assay (PEA) technology, which is a sensitive, quantitative method to examine protein abundance in complex fluids, such as plasma, and EVs^31–34^. EV inflammatory proteins that met our threshold criteria (see Materials and Methods) are listed in Supplementary Table 2. Linear regression was used to analyze the relationship between the normalized protein levels (NPL) and mortality status controlling for sex, race, and poverty status. The EV inflammatory proteins chemokine (C-C motif) ligand 23 (CCL23), macrophage-colony stimulating factor (CSF-1), chemokine (C-X-C motif) ligand 9 CXCL9, glial-cell-line-derived neurotrophic factor (GDNF), monocyte chemoattractant protein-1 (MCP-1), STAM-binding protein (STAMBP), and eukaryotic translation initiation factor 4E (eIF4E)-binding protein 1 (4E-BP1) and were found to be significantly associated with mortality (Fig. 3 and Table 2). These EV inflammatory proteins were all significantly higher in individuals who died within five years versus those who remained alive.

**Table 2:** EV inflammatory proteins significantly associated with mortality, poverty status, race, and sex.

Protein symbol	Mortality	Poverty status	Race	Sex	ExoCarta/Vesiclepedia	Protein function	Reference
4E-BP1	x		x		Yes	RNA metabolism	^46^
CCL4			x			Chemokine; chemoattractant of immune, endothelial and other cells	^47^
CCL11			x			Chemokine; immune response in inflammatory-related and allergic diseases	^48^
CCL19			x			Chemokine; lymphoid organization, immune response initiation, immune cell trafficking	^49^
CCL23	x		x			Chemokine; chemoattractant of immune cells in tumors	^39^
CD5				x	Yes	Scavenger receptor; immunomodulatory function, pattern recognition receptors	^50^
CD8A				x	Yes	Cell surface receptor on cytotoxic T cells; mediates immune cell–cell interactions	^51^
CSF-1	x				Yes	Cytokine; differentiation, proliferation, migration, function of myeloid progenitor cells	^37^
CST5		x	x		Yes	Type 2 cystatin; cysteine proteinase inhibitor	^52^
CXCL5			x			Chemokine; promotes angiogenesis and tumorigenesis, remodels connective tissue	^53^
CXCL6				x		Chemokine; recruits neutrophils for anti-microbial actions	^54^
CXCL9	x	x				Chemokine; immune cell differentiation, migration and activation	^38^
CXCL10		x				Pro-inflammatory chemokine; inflammation and response to infection	^38^
CXCL11		x	x	x		Pro-inflammatory chemokine; inflammation and response to infection	^38^
DNER			x		Yes	Neuron-specific receptor for Notch; promotes tumorigenesis in other tissues	^55^
FGF-19			x		Yes	Endocrine factor; metabolism, protein synthesis, carcinogenesis	^56^
GDNF	x					Neurotrophic factor; neuroinflammation	^40^
IL-12B		x	x	x		Cytokine; sustains immune responses against pathogens and other immune functions	^57^
MCP-1	x					Chemokine; potential marker of biological age	^36^
MCP-2			x			Chemokine; promotes cancer cell proliferation and migration	^39^
MMP-1			x		Yes	Breaks down extracellular matrix proteins in physiological and pathological processes	^58^
OPG			x			Secreted receptor in TNF superfamily; bone remodeling and homeostasis	^59^
SCF			x			Binds to c-Kit receptor and regulates hematopoiesis and melanogenesis	^60^
STAMBP	x				Yes	Regulates intracellular cargo trafficking in the endosomal pathway	^61^
VEGFA		x	x		Yes	Mitogen that acts on endothelial cells and promotes vasculogenesis, angiogenesis	^62^

Proteins significantly associated with mortality, poverty status, race, and sex were determined from linear regressions and indicated with an X in the columns. Proteins reported to be present in EVs in databases (ExoCarta and Vesiclepedia) are indicated with a “yes”. General functions of proteins are listed, not necessarily functions attributed to these proteins in EVs.

**Figure 4:**
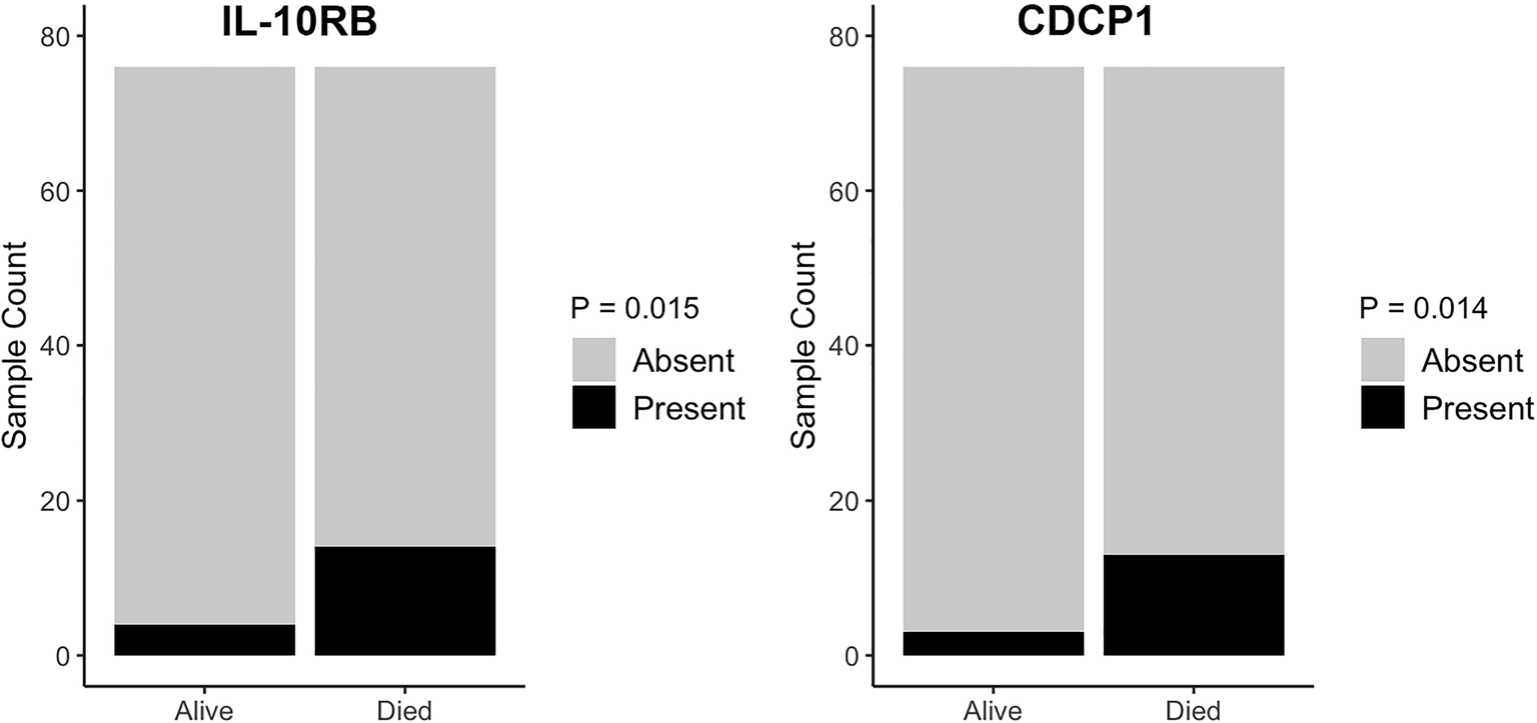
The presence of EV IL-10RB and CDCP1 and mortality. Plasma EVs were isolated from 76 individuals deceased within 5 years along with 76 surviving individuals and lysed EVs were analyzed using a proximity extension assay. Presence of each inflammatory protein with mortality status was analyzed using logistic regression accounting for sex, race and poverty status.

We also examined all 92 EV proteins in terms of presence or absence to determine if the presence, but not amount, of the protein was associated with mortality status. Logistic regression analyses indicated that the presence of interleukin-10 receptor subunit beta (IL-10RB) and CUB domain-containing protein 1 (CDCP1) were significantly associated with mortality status, controlling for sex, race, and poverty status (Fig. 4). Specifically, IL-10RB and CDCP1 were more likely to be present in EVs from individuals who had died within five years, than in their alive counterparts (Fig. 4).

### Plasma EV inflammatory proteins are associated with race, sex, and poverty status

**Figure 5:**
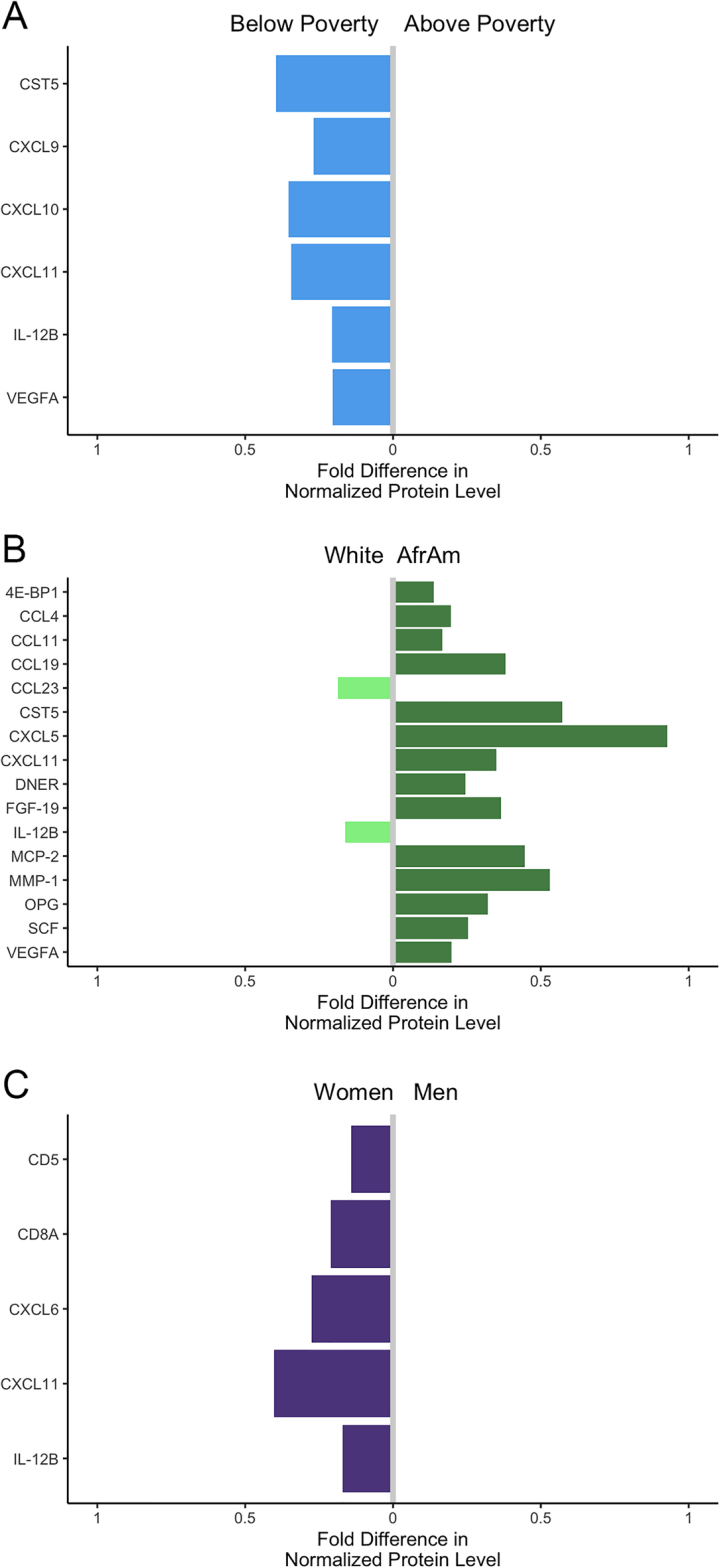
Plasma EV protein levels are altered with poverty status, race, and sex. Plasma EVs were isolated from 76 individuals deceased within 5 years along with 76 surviving individuals (Table 1). EVs were lysed and analyzed using a proximity extension assay. Mean differences in Normalized Protein Levels are shown in log_2_ scale and are interpreted as fold differences. Proteins significantly associated with poverty status (**A**) race (**B**) and sex (**C**) were determined from linear regressions with all variables (sex, race, poverty status, mortality status) in the model.

Previously, we found that EV proteins were associated with clinical markers of mortality and this relationship depended on race^25^. We also reported a higher EV level of phospho-IGF-1R in women compared to men. In addition, little is known about differences in EV cargo with various demographics^35^. Here, we wanted to examine if there were differences in EV inflammatory proteins with race, sex, and poverty status. Linear regression accounting for the study design of race, sex, poverty status, and mortality status in the model was used to assess the association of each demographic variable with each protein. CST5, CXCL9, CXCL10, CXCL11, IL-12B and VEGFA were all significantly higher in individuals living below poverty compared to those above poverty (Fig. 5A and Table 2). Furthermore, there were many EV inflammatory proteins that were different between African American and White participants (Fig. 5B and Table 2). Except for CCL23 and IL-12B, the levels of these EV inflammatory proteins were higher in African American participants than White participants. Five EV-associated proteins, CD5, CD8A, CXCL6, CXCL11, and IL-12B, were significantly higher in women compared to men (Fig. 5C and Table 2).

## Discussion

Although there are well-established markers of mortality, little is known about whether EVs and their associated cargo are biomarkers of mortality. Here we analyzed plasma EV characteristics including, concentration, size, and associated cargo in a cohort of racially diverse African American and White participants who had either died within 5 years of donating a plasma sample and their matched surviving controls. We found no significant changes in plasma EV concentration or EV mtDNA levels between these two groups. However, we report a significant difference in EV inflammatory protein levels in EVs isolated from individuals who died in the ensuing 5 years. These data suggest that the EV protein cargo may be potential markers of future mortality within a defined period.

Most biomarker mortality studies have been conducted in elderly, mostly White cohorts. Our cohort consisted of African American and White adults who were between 45 and 74 years old (mean age ~ 59 years) at the time of their plasma sample. This age range allowed for the identification of markers that may identify individuals at risk for early mortality. Given the disparities in life expectancies associated with minority status and socioeconomic status, we designed our cohort to consist of both African American and White adults living above and below poverty. Therefore, we could also examine whether poverty status, race, or sex influenced the presence or level of EV-associated inflammatory proteins.

We found significantly higher EV levels of CCL23, CSF-1, CXCL9, GDNF, MCP-1, STAMBP, and 4E-BP1 among those individuals in the mortality cohort. Higher levels of these inflammatory proteins may indicate a heightened level of systemic inflammation. Inflammation is a contributor to age-related diseases such as cancer and cardiovascular disease. Here, we chose participants with confirmed death from cancer or cardiovascular disease as these diseases represent the most common causes of deaths for HANDLS participants^26^ and the US nationally^27^. These data indicate that these EV inflammatory proteins may be indicators of significant risk for cardiovascular or cancer-related deaths within a 5 year period.

It remains to be determined whether these EV inflammatory proteins are indicators of specific pathologies or if they are general markers of inflammation. Existing data suggests that circulating levels of MCP-1 might be a biomarker of age and health in mice and that MCP-1 levels increased in frail individuals who are at risk for mortality^36^. Our data supports the idea that MCP-1 is a biomarker of mortality as well as a possible measure of biological age.

CSF-1 is critical in the differentiation of myeloid progenitors and regulates the proliferation, migration, and function of peripheral macrophages^37^. Higher circulating levels of CSF-1 are found in various pathologies including chronic inflammatory disease, cancer, and infections. CXCL9 and CCL23 are both chemokines. CXCL9 regulates immune cell differentiation, migration and activation and has been implicated in various diseases including inflammatory diseases and cancer^38^. CCL23 has been shown to have a limited role in cancer but may act as a chemoattractant of immune cells to regulate bone and vascular remodeling in the tumor niche^39^. Thus, it can be speculated that higher levels of these cytokines and chemokines on EVs may reflect underlying inflammatory conditions that may promote tumorigenesis.

GDNF is an important neurotrophic factor that acts on neurons but also plays a critical role in other tissues including the kidney^40^. Due to its role in promoting survival of dopaminergic neurons, it has been pursued as a possible treatment for Parkinson’s disease^40^. However, GDNF also may be upregulated during neuroinflammation and thus may play either a detrimental of beneficial role depending on cellular context and its role in plasma EVs is not known.

4E-BP1 and STAMBP are both intracellular proteins. Little is known about 4E-BP1 in EVs. Previously, we detected this protein in plasma EVs in a cohort of euglycemic and diabetic individuals^31^. STAMBP (also known as AMSH, associated molecule with the SH3 domain of STAM) regulates intracellular cargo trafficking in the endosomal pathway through its deubiquitination activities on the endosomal sorting complexes required for transport (ESCRTs) proteins. This interaction can modulate ubiquitination and sorting of protein cargo. Although STAMBP has previously been identified in EVs (Table 2; www.vesiclepedia.com;^31^), there is limited information about its role as EV cargo.

IL-10RB and CDCP1 were more likely to be present in EVs from individuals in the mortality cohort than in their alive counterparts. IL-10RB and CDCP1 are both transmembrane proteins that plays important cell signaling roles in cells. IL-10RB levels in EVs were previously reported to be associated with HOMA-IR and HOMA of β-cell function^31^. CDCP1 has been identified in prostate cancer-EVs, indicating the presence of this cell surface protein on EVs may be a biomarker for cancer^41^.

Little is known about the biological consequences of social determinants of health on EVs and their associated cargo. Since we designed our cohort to consist of participants living above and below poverty, we were able to discover that CST5, CXCL9, CXCL10, CXCL11, IL-12B and VEGFA were all significantly higher in EVs from individuals living below poverty compared to those living above poverty. CXCL9/10/11 belong to a family of chemokines that bind and signal through the CXCR3 cell surface receptor. These interferon-gamma induced chemokines are secreted by different immune cells and from other cell types^38^. These proinflammatory chemokines are involved in inflammation and response to infection and have been implicated in a variety of diseases including autoimmune, inflammatory, infectious as well as in cancer^38^. It is well-known that those living in poverty have higher rates of chronic infections such as cytomegalovirus (CMV) infection^42^ and autoimmune diseases^43^. In agreement with this finding, IL-12B also plays an important role in sustaining immune responses against pathogens. In addition, there are well documented gene expression changes with adversity^8^. The Conserved Transcriptional Response to Adversity is a pattern of gene expression characterized by up-regulation of pro-inflammatory genes and down regulation of genes involved in interferon mediated innate antiviral responses^44,45^. It is possible that poverty induced increased gene expression may result in the presence of these EV inflammatory proteins in the circulation to help combat chronic infection that is prevalent in individuals living below poverty.

Few studies have examined whether race influences EVs^35^. Previously, we reported that there were racial differences in EV protein cargo and found higher levels of phospho-p53, total p53, cleaved caspase 3, ERK1/2 and phospho-AKT in White individuals compared to African American individuals^25^. EV concentration was significantly associated with several clinical mortality risk factors and the relationship between several EV proteins with mortality markers were dependent on race. Here, we found that 16 different EV inflammatory proteins were significantly different between African American and White participants. Except for CCL23 and IL-12B, the levels of these EV inflammatory proteins were higher in African American compared to White individuals. We also examined differences between men and women and found that five EV inflammatory proteins, CD5, CD8A, CXCL6, CXCL11, and IL-12B were higher in women than men. In Table 2, we list general functions of all the proteins identified in our analysis to provide additional information about these inflammatory proteins.

It may be surprising that there were no significant differences in EV mtDNA levels with mortality status. However, there are only few studies that have examined mtDNA in plasma EVs^30,63,64^. Previously, we reported that EV mtDNA levels decline with advancing age^28^. Future research lies in examining mtDNA in plasma EVs and in which pathologies and conditions that this cargo act as biological indicators of health and disease.

There are several limitations to our study. Our cohort size is smaller than most epidemiological studies that examine clinical mortality markers. However, this cohort size is similar to most EV studies using plasma or other biofluids^28,65–68^. Here we are utilizing a precipitation approach to separate our EVs. This approach allows for a high-throughput approach for EV isolation while also maintaining a reproducible technique with a low coefficient of variance^28^. In addition, this method can isolate EVs from lower amounts of plasma (0.5 ml) and enables us to examine multiple downstream analyses of EVs and associated cargo. Each different EV isolation technique has its own advantages and disadvantages, and here we cannot exclude that some various molecules (i.e. soluble plasma proteins or lipoproteins) may be precipitated along with the EVs in our isolation process. Currently, it remains a challenge in the field to fully separate EVs from lipoproteins in plasma^69^, which may partially be because lipoproteins can associate with EVs^70^. In addition, we have used stringent criteria to include proteins in our analysis (> 70% detectable in our samples) and detected both intracellular and extracellular proteins. Furthermore, cytokines and chemokines have been reported to be EV-associated, which may affect biological activity and function^10,71^.

In conclusion, we have identified that there are several different inflammatory proteins that are higher in EVs from individuals that later died compared to their surviving controls. These data may provide further insight into the processes that underlie early mortality and may help guide the further development of biomarkers for midlife mortality.

## Methods

### Clinical study participants

The study cohort was selected from the Healthy Aging in Neighborhoods of Diversity across the Life Span (HANDLS) study of the National Institute on Aging Intramural Research Program, National Institutes of Health (NIH). HANDLS is an epidemiologic, longitudinal study of health disparities and aging, examining the effects of race and socioeconomic status in community-dwelling older adults^72^. Participants undergo physical exams and structured medical history interviews, complete questionnaires and contribute blood samples approximately every five years. HANDLS is approved by the Institutional Review Board of the National Institutes of Health. All participants provided written informed consent. All experiments were performed in accordance with relevant guidelines and regulations. Race is self-identified as African American or White. Participants were either above or below poverty as defined by 125% of the 2004 U.S. Health and Human Services Poverty Guidelines at enrollment. Prospective mortality and cause of death (International Classification of Disease, ICD, 10) is from linked National Death Index data^73^ for study participants from enrollment (August 2004–March 2009) through December 31, 2017.

The cohort was designed to compare participants with confirmed death from cancer (ICD10 code 'C') or cardiovascular disease (ICD10 code ‘I’) within five years of a fasting plasma sample to those alive through December 31, 2017. Cancer and cardiovascular disease are the most common causes of deaths for HANDLS participants^26^ and the US nationally^27^. HIV positive participants were excluded, and all participants were between 45 and 74 years old at the time of their blood sample. Eighty participants who died within five years were randomly selected across the factorial design of sex, race (African American or White) and poverty status (above or below) and a randomly selected comparison group from those alive through 2017 was selected matched on sex, race, poverty status and 10 year age groups. Due to plasma availability, the final sample consisted of 152 participants; 76 who died within five years of the selected fasting plasma sample, the mortality cohort, and 76 living participants, the alive cohort. White participants living below poverty are underrepresented.

### Plasma EV isolation

EVs were isolated from 0.5 ml plasma using ExoQuick™ Exosome Precipitation Solution (System Bioscience Inc.) as previously published^28^. Previously, we reported that this separation method had the lowest coefficient of variance compared to other isolation techniques including ultracentrifugation and size exclusion columns and was also more practical for isolating EVs from a large number of samples^28^.

Plasma (0.5 ml) aliquots were treated with 0.2 ml Thromboplastin D (Cat#:100,354; Fisher Scientific, Inc.), and incubated at room temperature for one hour. Dulbecco’s phosphate buffered saline (DPBS^-2^) (0.3 ml) with 3 × concentrated protease and phosphatase inhibitor cocktails (Roche Applied Sciences) was added. Samples were then centrifuged at 3000* g* for 20 min at 4 °C. ExoQuick™ Exosome Precipitation Solution (252 µl; System Bioscience Inc.) was added and incubated at 4 °C for one hour. The samples were subsequently centrifuged at 1500* g* at 4 °C for 20 min. The supernatant was separated and saved for analysis as the EV depleted plasma fraction. The EV pellet was resuspended in 0.5 ml of DPBS^−2^ with 3 × concentrated protease and phosphatase inhibitor cocktails (Roche Applied Sciences). Isolated EVs were stored at  − 80 °C prior to their use.

### Transmission electron microscopy

Electron Microscopy imaging was completed by the Johns Hopkins University Neurology Microscopy Core as previously published^28,66^. EVs were adsorbed to freshly ionized 300 mesh formvar/carbon coated grids then washed briefly through 5–7 puddles of ddH2O and then negatively stained in 2% aqueous uranyl acetate. The grids were visualized on a Libra 120 Transmission Electron Microscope at 120 kV (Zeiss). A Veleta camera (Olympus) was used to acquire images.

### ELISA

Plasma EV samples were lysed (1:3) in Mammalian Protein Extraction Reagent (MPER™) supplemented with protease and phosphatase inhibitors (Roche). Equal concentrations of EV lysates were used to quantitatively measure the EV markers CD81 (CSB-EL004960HU) and ALIX (CSB-EL017673HU) using kits from CUSABIO according to manufacturer’s instructions.

### Exo-Check™ exosome antibody array

Exo-Check™ Exosome Antibody Array (System Biosciences; EXORAY200B) was used to assess the presence of the EV markers: CD63, CD81, ALIX, FLOT1, ICAM1, EpCam, ANXA5 and TSG101. Plasma EVs were used in the array according to manufacturer's instructions.

### Nanoparticle tracking analysis

Isolated plasma EV samples were diluted 1:300 in filtered PBS. Nanoparticle tracking analysis (NTA) was used to quantify vesicle size and concentration on a NanoSight NS500 (Malvern Instruments Ltd.). Samples were recorded at camera level = 14, detection level = 4 for 5 videos of 20 s. The mean coefficient of variation for the EV concentrations of our samples used for analysis was 3.04%. Software NTA 3.3 Dev Build 3.3.104 was used. All samples were analyzed on the same instrument by the same operator.

### DNA isolation from plasma derived EVs

Plasma EV samples (50 µl) were first DNase treated (Lucigen, Cat: DB0715K) to degrade any DNA on the outside of the EVs. Samples were treated in a reaction containing 6.5 µl of DNase Reaction Buffer, and 5 U of DNase at 37 °C for 30 min. The reaction was stopped by the addition of 6.5 µl DNase Stop Solution at 65 °C for 10 min. Each sample was then equalized to 200 µl with the addition of 125 µl of nuclease free water and lysed by adding 20 µl of Proteinase k, which is included in the DNeasy Blood and Tissue kit (Qiagen, Cat: 69,506). The manufacturer’s DNA extraction protocol was then followed with additional modifications as indicated previously^30^. The eluted DNA (~ 50 µl) was diluted in an additional 50 µl of AE Buffer (Qiagen) and stored at − 20 °C.

### Quantitative real-time PCR

For quantitative real-time PCR (qPCR) analysis, each of our participant samples were run blinded to participant information. Duplicate samples were run, and the duplicate mean was used in subsequent analysis. Reactions (13 µl) were performed with mitochondrial gene-specific primers (2.5 µl/rxn), TaqMan™ Fast Advanced Master Mix (7.5 µl/rxn) and 3 µl of DNA per reaction. The primer sets used were previously described^30^ and the sequences are listed in Supplementary Table 1. qPCR was performed on an Applied Biosystems 7900HT Fast Real-Time PCR System. The thermal profile for mtDNA qPCR was as follows: 3 min at 95°C followed by 40 cycles of 20 s at 95 °C and 20 s at 60 °C.

We calculated the relative level of each mtDNA primer set using a derivation of the 2^−ΔΔCt^ method. Values were normalized to the global mean (X; mean of the Ct for each primer set) using this formula 2^−(X−Ct)^^74^. We used this method since a reliable internal control could not be amplified from DNA isolated from plasma EVs. We tried to utilize several reported normalization genes, but these genes did not consistently amplify in the different samples and also the amplicon efficiencies of the mtDNA and references were not equal as recommended for using a proper reference value^74^. The EV mtDNA values were skewed and thus were log_2_ transformed.

### Olink proteomics

Plasma EVs from each of the participants were lysed in MPER (1:3) and then protein concentration was analyzed using a Bradford assay. Equal amounts of lysed EVs (56.7 μg total protein in 40 μl final volume of PBS; f.c. 1.4 g/μl) were aliquoted and analyzed by the Olink® Proteomics biomarker Inflammation Panel via their Multiplex Proximity Extension Assay (PEA). PEA allows for the high throughput, precise detection and quantification of multiple biomarkers. Assays were run blinded to experimental groupings by Olink® Proteomics. Multiple internal and external controls were utilized throughout for quality control and for data normalization. Protein data were normalized and reported in Normalized Protein eXpression units which are on a log_2_ scale, here referred to as normalized protein levels (NPL). In total, 92 proteins were tested using the Olink® Inflammation panel. Of those 92 proteins, 39 met our threshold of being detected in > 70% of the samples and their NPL values were utilized for further analysis. These 39 proteins are listed in Supplementary Table 2. Proteins with skewed distributions were log_2_-transformed for statistical analysis (CCL4, CST5, CXCL6, MCP-1, STAMBP, 4E-BP1). Due to negative NPL values, CST5 was shifted (+ 2) before transformation. All 92 proteins were used in the presence/absence analyses.

## Statistics

Statistical analysis was performed using R software version: R 4.0.3 ^75^. Student’s t-test was used to assess the differences between the groups for age and EV marker levels, while Pearson’s chi-squared test was used for sex, race, and poverty status. Correlations among variables of interest were assessed by Pearson correlation. EV protein levels, mtDNA levels and EV concentration were analyzed using linear regression which modeled the study design of mortality status, sex, race and poverty status. Statistical significance was based on significance of the relevant coefficient in the model. Presence of EV proteins were analyzed using threshold of detection data via logistic regression with mortality status, sex, race and poverty status.

## Data availability 

The datasets generated and analyzed during the current study are available from the corresponding author on reasonable request through the HANDLS website https://handls.nih.gov/.

## Supplementary Information

Supplementary Information.
