# Supplementary material for: Association of extracellular vesicle inflammatory proteins and mortality
Source: Sci Rep. 2022 Aug 18;12:14049. doi: 10.1038/s41598-022-17944-z (PMC9386667; doi:10.1038/s41598-022-17944-z)
Supplement: Supplementary file 1 — Supplementary Information. [file 41598_2022_17944_MOESM1_ESM.pdf]

Supplementary Information

Association of Extracellular Vesicle Inflammatory Proteins and Mortality

Nicole Noren Hooten, Stephanie Torres, Nicolle A. Mode, Alan B. Zonderman, Paritosh Ghosh, Ngozi Ezike and Michele K. Evans

Supplementary Table 1. Primer Sequences for qPCR

Gene	Primer Name	Forward Sequence	Reverse Sequence	Probe	Ref.	Size (bp)
MT-RNR2/ MT-TL1	Mito_3164	5'CCTTCCCCCGTAAATGATATCA3'	5'GCCATCTTAACAAACCCTGTTCTT3'	5’FAM-AACTTAGTATTATACCCACACCC-MGB3'	1	76
MT-ND2	Mito_4625	5'CACAGAAGCTGCCATCAAGTA3'	5'CCGGAGAGTATATTGTTGAAGAG3'	5’FAM-CCTCACGCAAGCAACCGCATCC-BLACK HOLE-3'	2	89
MT-COX2	Mito_7878	5´AATCAATTGGCGACCAATGG3´	5´CGCCTGGTTCTAGGAATAATGG3´	5’FAM-ACTGAACCTACGAGTACAC-MGB-3’	3	100
MT-ATP8	Mito_8446	5’AATATTAAACACAAACTACCACCTACCT3’	5’TGGTTCTCAGGGTTTGTTATAA3’	5’-FAM-CCTCACCAAAGCCCATA-MGB-3’	4	79

Supplementary Table 2. Inflammatory proteins detected in plasma EVs.

Protein Symbol	Protein Name
CCL11	Eotaxin-1
CCL19	C-C motif chemokine 19
CCL23	C-C motif chemokine 23
CCL28	C-C motif chemokine 28
CCL4	C-C motif chemokine 4
CD244	Natural killer cell receptor 2B4
CD40	CD40L receptor
CD5	T-cell surface glycoprotein CD5
CD8A	T-cell surface glycoprotein CD8 alpha chain
CSF-1	Macrophage colony-stimulating factor 1
CST5	Cystatin D
CXCL5	C-X-C motif chemokine 5
CXCL6	C-X-C motif chemokine 6
CXCL9	C-X-C motif chemokine 9
CXCL10	C-X-C motif chemokine 10
CXCL11	C-X-C motif chemokine 11
DNER	Delta and Notch-like epidermal growth factor related receptor
FGF-19	Fibroblast growth factor 19
Flt3L	Fms-related tyrosine kinase 3 ligand
GDNF	Glial cell line-derived neurotrophic factor
HGF	Hepatocyte growth factor
IL-12B	Interleukin-12 subunit beta
IL18	Interleukin-18
IL-18R1	Interleukin-18 receptor 1
LAP-TGF-beta-1	Latency-associated peptide transforming growth factor beta-1
MCP-1	Monocyte chemotactic protein 1
MCP-2	Monocyte chemotactic protein 2
MCP-4	Monocyte chemotactic protein 4
MMP-1	Matrix metalloproteinase-1
MMP-10	Matrix metalloproteinase-10
OPG	Osteoprotegerin
SCF	Stem cell factor
STAMBP	STAM-binding protein
TGF-alpha	Transforming growth factor alpha
TRAIL	TNF-related apoptosis-inducing ligand
TWEAK	Tumor necrosis factor (Ligand) superfamily, member 12
uPA	Urokinase-type plasminogen activator
VEGFA	Vascular endothelial growth factor A
4E-BP1	Eukaryotic translation initiation factor 4E-binding protein 1

Supplementary Figure 1

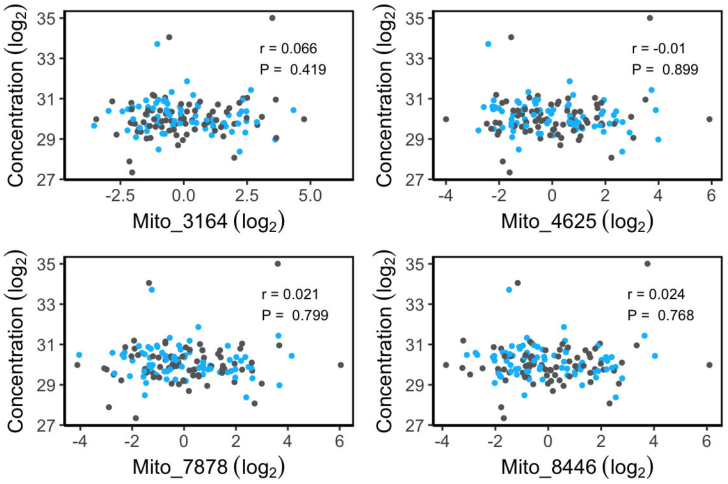

Supplementary Figure 1. mtDNA levels do not correlate with EV concentration. Plasma EVs were isolated from 76 individuals deceased within 5 years along with 76 surviving individuals. DNA was isolated and EV mtDNA levels (log2 transformed) were measured using mtDNA specific primers from four different regions of the mitochondrial genome using qPCR. EV concentration was measured using nanoparticle tracking analysis and was log2 transformed for analysis. Pearson correlation r values and P values are indicated.
